# Physical Activity, Immune System, and the Microbiome in Cardiovascular Disease

**DOI:** 10.3389/fphys.2018.00763

**Published:** 2018-06-29

**Authors:** Dawn M. Fernandez, Jose C. Clemente, Chiara Giannarelli

**Affiliations:** ^1^Department of Medicine, Cardiovascular Research Center, Icahn School of Medicine at Mount Sinai, New York, NY, United States; ^2^Department of Genetics and Genomic Sciences, Icahn School of Medicine at Mount Sinai, New York, NY, United States; ^3^Precision Immunology Institute, Icahn School of Medicine at Mount Sinai, New York, NY, United States

**Keywords:** atherosclerosis, inflammation, microbiome, cardiovascular disease, physical activity

## Abstract

Cardiovascular health is a primary research focus, as it is a leading contributor to mortality and morbidity worldwide, and is prohibitively costly for healthcare. Atherosclerosis, the main driver of cardiovascular disease, is now recognized as an inflammatory disorder. Physical activity (PA) may have a more important role in cardiovascular health than previously expected. This review overviews the contribution of PA to cardiovascular health, the inflammatory role of atherosclerosis, and the emerging evidence of the microbiome as a regulator of inflammation.

## An Overview of Atherosclerotic Cardiovascular Disease

Cardiovascular disease (CVD) is the worldwide leading cause of death and is a global economic burden (Cook et al., 2014). Most of the disease is driven by a process known as atherosclerosis—the buildup of plaque which occludes arterial vessels (Rajamani and Fisher, 2017). Throughout disease progression, the atherosclerotic plaque loses stability and becomes prone to rupture, a sudden event that can lead to arterial thrombosis and cause deleterious acute ischemic events like myocardial infarction (MI) and stroke (Gisterå and Hansson, 2017).

Atherosclerosis is influenced by a number of risk factors including, lifestyle choices (i.e., diet, physical activity, and cigarette smoking), advancing age, and associated disorders like hypertension, diabetes, obesity, and dyslipidemia (Tegos et al., 2001). However, traditional risk factors alone are inadequate at predicting atherosclerotic CVD. According to the Participants of Early Subclinical Atherosclerosis (PESA) study, subclinical atherosclerosis was detected in almost 50% of participants that were free of the conventional cardiovascular risk factors (Fernández-Friera et al., 2017). Statins, the gold standard treatment for lowering lipids (Grundy, 2016), have proven effective at reducing cardiovascular events, yet their contribution to reducing mortality remains questionable (Cholesterol Treatment Trialists’ [CTT] Collaborators, 2012; DuBroff and de Lorgeril, 2015). The recently developed proprotein convertase subtilisin-kexin type 9 (PCSK9) inhibitors effectively lowered cholesterol levels and reduced cardiovascular events when taken in conjunction with statins (Waters and Hsue, 2017). However, some of these studies failed to meet expectations based on the linear relationship between LDL reduction and percentage reduction of cardiovascular events based on data from 14 clinical trials using statins (Waters and Hsue, 2017). A possible explanation is that PCSK9 inhibitors failed to reduce C- reactive protein (CRP) levels, a clinical biomarker of inflammation and cardiovascular risk (Waters and Hsue, 2017).

Atherosclerosis is recognized as a chronic systemic inflammatory disease with focal manifestations at the vascular site (Ross, 1999; Conti and Shaik-Dasthagirisaeb, 2015). CVD is often pronounced in other immune disorders such as rheumatoid arthritis (RA) or systemic lupus erythematosus (SLE) (Gabriel, 2008; Abu-Shakra and Novack, 2012; Skeoch and Bruce, 2015; Henrot et al., 2018). Recent studies propose the gut microbiome as an emerging regulator of inflammatory conditions including atherosclerosis (Ridaura et al., 2013; Romano et al., 2015; Halfvarson et al., 2017; Kreznar et al., 2017; Slingerland et al., 2017).

The World Health Organization, American Heart Association, and European Society of Cardiology recommend several significant behavioral changes for the preventative care of CVD including smoking cessation, dietary changes, weight control, alcohol intake, and physical activity (PA) (Goff et al., 2014; Piepoli et al., 2016; Whelton et al., 2018). Of particular interest is the contribution of PA to CVD (Lin et al., 2015; Murtagh et al., 2015). Utilizing PA as therapy is particularly appealing because its implementation is cost-effective for the patient and the benefits can ease the global economic burden by reducing the cost of care (Valero-Elizondo et al., 2016). Recently, PA has been the focus of intense investigation for its ability to regulate the underlying immune system. This review will focus on the impact of PA as it relates to the regulation of systemic inflammation and the contribution of the microbiome.

## Physical Activity and Cardiovascular Health

Regular PA is associated with many health benefits, including improving cholesterol levels, reducing body weight and blood pressure, increasing insulin sensitivity, and neuroprotective effects (Myers, 2003; Chieffi et al., 2017). Indeed, some of these benefits have been attributed to in part by the small neuropeptides called Orexins (Chieffi et al., 2017). Orexin A is released into plasma upon exercise, and contributes to regulating energy balance (Messina et al., 2016, 2017).

Numerous studies have established that PA is beneficial for reducing the risk and effect of CVD (Diaz et al., 2016; Vella et al., 2017; Florido et al., 2018). Physically fit individuals have a reduced risk of developing CVD (Kokkinos, 2012). A 10 year follow up study that surveyed senior participants found that leisure time PA reduced the risk of CVD incidents and mortality in a dose-dependent manner (Barengo et al., 2017). The Multi-Ethnic Study of Atherosclerosis (MESA) suggests that moderate to vigorous PA accompanies a more favorable inflammatory marker profile (Vella et al., 2017). The Atherosclerotic Risk in Communities (ARIC) Study evaluated participants with no history of CVD, and found that maintenance and engagement of PA were effective in decreasing heart failure risk (Florido et al., 2018). Additionally, the HUNT Study (Nord-Trondelag Health Study) showed that sustained PA, and not weight loss, substantially improved survival at 30 years follow up in individuals with coronary artery disease (Moholdt et al., 2018).

Physical activity has profound effects on vascular function and lumen dimension, and structural cardiac modifications (Green and Smith, 2017; Francavilla et al., 2018). For example PA regulates heart rate variability (HRV), a predictive factor for sudden cardiac death and MI (Sessa et al., 2018). Elderly athletes display less Carotid Intima Thickening and a more favorable HRV compared to their sedentary counterparts (Galetta et al., 2013; Soares-Miranda et al., 2016). Animal models have also demonstrated that old rats subjected to exercise training had reversed age-related microvascular dysfunction (Hotta et al., 2017).

Sedentary behavior (SB) is described as the lowest energy expenditure for waking activities (e.g., sitting or lying down), and is measured by metabolic equivalents (METs). Although uncoupling SB from associated illness and other risk factors like obesity and RA is difficult, SB promotes a pro-inflammatory status (Fenton et al., 2017). Biomarker analysis from a cross-sectional study of senior men found that higher levels of SB correlated with higher levels of pro-inflammatory markers IL-6, CRP, and tPA (Parsons et al., 2017). There is evidence that reallocation of SB with moderate to vigorous PA promotes a better inflammatory profile, with increased adiponectin levels and lower IL-6, C3, leptin, and leukocyte concentrations (Phillips et al., 2017). Moreover, patients with an inflammatory disease like RA, that have extended sedentary bouts have an increased risk of developing cardiovascular events and could benefit by interrupting sedentary time with leisurely PA (Fenton et al., 2017). Long-term studies, like the 15-years long Tanushimaru Study, confirm that decreased sitting time reduces the risk of mortality (Sakaue et al., 2017).

## Inflammation and Atherosclerosis

The first critical step of atherosclerosis development is endothelial dysfunction and increased endothelial permeability that facilitates the build-up and deposition of low-density lipoproteins (LDLs) into the intima layer of the arterial wall where they become oxidized (oxLDL) (Lusis, 2000). Monocytes from the circulation infiltrate the arterial wall and differentiate into macrophages that engulf oxLDL becoming foam cells. Foam cells trapped in the intima layer become apoptotic and necrotic, which form the basis of a necrotic core (Ross, 1999; Gisterå and Hansson, 2017). Immune cells express cytokines and chemokines that are critical modulators of inflammatory signaling during atherogenesis (Turner et al., 2014; Ramji and Davies, 2015). Cytokines are also highly regulated during and as a consequence of exercise (Pedersen, 2017). Although there are many cytokines implicated in atherosclerosis, we will address the top key players, which are summarized in **Table 1**.

**Table 1:** Regulation of key chemokines and cytokines in atherosclerosis and physical activity.

Cytokine	CVD effect	Regulation in atherosclerosis	Reference	PA effect	Regulation in physical activity	Reference
CCL2/MCP-1	↑	Upregulated in atherosclerosis	Lin et al., 2014	**↓**	Low intensity training for 8 weeks decreased mRNA levels of MCP-1 in leukocytes	Yakeu et al., 2010
CCL5	↑	Blocking receptor binding reduces Atherosclerotic Plaque Formation	Veillard et al., 2004	**↓**	Circulating CCL5 was decreased in obese patients subjected to 3 months of physical activity	Baturcam et al., 2014
CX3CL1	↑	Upregulated on Monocytes from Coronary Artery Disease patients	Apostolakis et al., 2007	↑	Increased after a single bout of exercise	Strömberg et al., 2016
IFN- γ	↑	Induces macrophage gene expression. Mice lacking IFN-gamma receptor have reduced atherosclerotic plaque	Gupta et al., 1997	↑	Moderate exercise increased levels on mononuclear cells	Zamani et al., 2017
IL-10	**↓**	Overexpression inhibits plaque progression in mice and decreases cholesterol levels	Eefting et al., 2007	↑	Increased by 940% on mononuclear cells in high-risk CVD patients subjected to long term exercise	Smith et al., 1999
IL-1b	↑	Inhibition decreases severity of atherosclerosis in mice and in humans	Kirii et al., 2003; Taylor et al., 2011; Shah et al., 2018	↑	Plasma concentrations increase immediately following exercise and remain elevated for 24 h	Moldoveanu et al., 2000
IL-2	↑	Blocking antibodies reduce atherosclerosis	Upadhya et al., 2004	**↓**	Levels decrease following strenuous exercise	Shephard and Shek, 1994
IL-4	↑	Conflicting Reports. IL-4 deficiency in mice reduces atherosclerosis, but exogenous delivery showed no involvement in the disease	Davenport and Tipping, 2003; King et al., 2007	↑	Increased by 94% on mononuclear cells in high-risk CVD patients subjected to long term exercise	Smith et al., 1999
IL-5	**↓**	Macrophage expression of IL-5 in mice reduced lesion size by 43%	Zhao et al., 2015	↑	Higher expression in the plasma profile of exercise trained individuals	Schild et al., 2016
IL-6	↑	Upregulated in cardiovascular disease. Exogenous expression of IL-6 increases plaque size	Huber et al., 1999	↑	100-fold increase after acute exercise	Pedersen et al., 2001
TGF-b	**↓**	Reduces atherosclerosis by weakening T cell activation	Robertson et al., 2003	↑	Increased by 43% on mononuclear cells in high-risk CVD patients subjected to long term exercise	Smith et al., 1999
TNF-α	↑	Inhibition reduces atherosclerosis in ApoE-/- mice	Branen et al., 2004	**↓**	Reduces circulating levels in patients with metabolic syndrome	Palomo et al., 2010

The initial injury to endothelial cells (ECs) by LDL typically occurs at the arterial branching points where laminar flow becomes disturbed, which results in morphological and functional changes that promote the permeability of the EC layer and allow retention of LDL (Moore et al., 2013). Activated ECs recruit immune cells by enhancing expression of adhesion molecules (e.g., VCAM and ICAM) and chemokines (Gimbrone and García-Cardeña, 2016). Pro-inflammatory chemokines are crucial for atherogenesis, for example, inhibition of a three chemokine axis (CCL2, CCL5, and CX3CL1) in mouse models leads to almost a complete attenuation of atherosclerosis (Ramji and Davies, 2015). Although chemokine pathways have been exploited as therapeutic targets (Sheikine and Hansson, 2006), few drugs have been FDA approved, but none for the treatment of atherosclerosis. For instance, the CCR5 antagonist Maraviroc was developed for the treatment of HIV and the low molecular weight CXCR4 antagonist Plerixafor (AMD3100) for stem cell mobilization (Koenen and Weber, 2011). In fact, modulating these pathways in complex disorders like CVD may be difficult due to the high risk of side effects like increased infection rate (Koenen and Weber, 2010).

Interferon-γ (IFN-γ) is a pro-inflammatory cytokine that functions as the primary activator of macrophages and has been shown to influence many stages of atherogenesis (Gupta et al., 1997; Voloshyna et al., 2014). IFN-γ enhances Monocyte Chemotactic Protein-1 (MCP-1) levels, a chemokine that recruits monocytes (Struyf et al., 1998) and the expression of Interferon-induced protein 35 (IFI35), a protein that contributes to EC proliferation and migration (Jian et al., 2018). Moreover, IFN-γ acts synergistically with other pro-inflammatory cytokines, like TNF-α to enhance chemokine production in monocytes and T-cells (Mehta et al., 2017).

Interleukin-1β (IL-1β) is a pro-inflammatory cytokine and a prime mediator of inflammation in CVD (Moss and Ramji, 2016). Therapeutic targeting of IL-1β in the Canakinumab Anti-inflammatory Thrombosis Outcomes Study (CANTOS) trial has provided the first proof-of-concept that inflammation is a key driver of cardiovascular events in high risk populations (Ridker et al., 2017). Inhibition of IL-1β reduced the risk of non-fatal CV events by 17% in patients with recent MI and elevated high sensitivity CRP (hs-CRP) (Ridker et al., 2017). Remarkably, a subgroup analysis of the CANTOS revealed that patients who achieved hs-CRP levels less than 2 mg/dl had a 25% reduction in major adverse cardiovascular events (Ridker et al., 2018), suggesting that the residual CV risk in this subpopulation was due to inflammation.

Interleukin-6 is a pleiotropic cytokine that is induced by IL-1β (Cahill and Rogers, 2008) and modulated by Canakinumab (Ridker et al., 2012). IL-6 can act as both a pro- or anti-inflammatory molecule, and thus its function is context dependent (Reiss et al., 2017). It participates in two distinct signaling mechanisms, binding to either the membrane-tethered IL-6 receptor (classical pathway) or a soluble form of the IL-6 receptor (trans-pathway). Trans-signaling is regarded as pro-inflammatory, while classical signaling is as anti-inflammatory (Scheller et al., 2011). Coronary artery disease patients have high levels of IL-6 which are also a predictive biomarker of the disease (Wainstein et al., 2017). However, mouse studies show that IL-6 depletion in ApoE-/- mice promotes atherosclerosis (Schieffer et al., 2004). Physical activity regulates IL-6 levels and acute exercise induces the release of IL-6 from the skeletal muscles into the circulatory system (Pedersen, 2017). Of note, IL-6 induced during exercise stimulates the release of anti-inflammatory cytokines IL-10 and IL-1ra (Pedersen, 2006, 2017). IL-6 is also a mediator of lipid metabolism that stimulates lipolysis and fat oxidation (van Hall et al., 2003) and has been shown to increase after a meal (Payette et al., 2009).

## Immune Cells and Physical Activity

Exercise has a profound effect on both the adaptive and innate immune system by regulating immune cell populations.

On the innate arm, natural killer (NK) cells, neutrophils, and monocyte are all regulated by exercise (Koelwyn et al., 2015). NK cells are modulated during exercise by increasing in number and cytotoxic activity (Pedersen and Ullum, 1994; Na et al., 2000) and increased NK cell infiltration of tumors was observed upon exercise (Pedersen et al., 2016). Neutrophils, a hallmark of acute inflammation, in aging become dysfunctional (Jackaman et al., 2017). One study showed that PA promotes the migratory function of neutrophils in older adults (Bartlett et al., 2016). PA can also influence monocyte polarization with a decrease of classical monocytes, and an increase of intermediate and non-classical monocytes (Slusher et al., 2017). Another study found that strenuous, anaerobic exercise leads to the acute mobilization of intermediate monocytes by a catecholamine-dependent mechanism (Steppich et al., 2000). Particularly, acute exercise was associated with a reduction of monocyte-platelets aggregates, which are associated with increased risk of cardiovascular clinical events (Michelson et al., 2001).

Physical activity also regulates the expression of inflammatory markers and this effect is dependent on the exercise intensity. For example, studies in obese individuals subjected to either medium or high intensity exercise regimes demonstrated that the training intensity can modulate differently the expression of chemokine receptors. Moderate training promoted expression of CCR2 and CXCR2 on monocytes, while higher intensity training promoted CCR5 expression on monocytes, neutrophils, and T-cells (Barry et al., 2017).

On the adaptive side, either prolonged or strenuous exercise affects the balance of types of T-cells. Exercise promotes the predominance of Th2 cells versus Th1 cells. Th1 cells produce IL-2 and IFN-γ, while Th2 cells produce IL-4, IL-5, IL-6, and IL-10 (Sharif et al., 2018). Regulatory T-cells (Treg) belong to a subset of T-helper cells, are anti-inflammatory, atheroprotective (Foks et al., 2015), and are also affected by exercise. A study of elite Olympic athletes from various disciplines, found that athletes displayed higher frequencies of Tregs compared to age and sex-matched controls and this effect was associated with PA intensity (Weinhold et al., 2016). Tregs release immunosuppressive cytokines like IL-10, IL-35, and TGF-β (Olson et al., 2013), and decrease the production of pro-inflammatory cytokines like IFN-γ (Mallat et al., 2003; Ou et al., 2018).

## The Microbiome and CVD: The Role of Physical Activity and Links to Inflammation

There is growing evidence indicating that the gut microbiome is a critical player in modulating host physiology (Clemente et al., 2012). Deviations from microbial homeostasis have been associated with various diseases, such as inflammatory bowel disease (Berg et al., 2015), arthritis (Clemente et al., 2018), or food allergies (Bunyavanich et al., 2016). Importantly, the microbiome has also been shown to play a critical role in obesity (Karlsson et al., 2012), atherosclerosis (Karlsson et al., 2012), and in the pathogenesis and progression of CVD (Kelly et al., 2016; Jie et al., 2017).

Karlsson et al. (2012) demonstrated that the genus *Collinsella* was enriched in patients with symptomatic atherosclerosis, while *Roseburia* and *Eubacterium* were enriched in healthy controls. These changes in bacterial composition were paralleled by enrichment in patients of bacterial genes encoding peptidoglycan synthesis (which might contribute to atherosclerosis by enhancing neutrophil function) and depletion of phytoene dehydrogenase and serum levels of b-carotene (hypothesized to have beneficial health effects) (Karlsson et al., 2012). A more recent study examined the association between gut microbiota and lifetime CVD risk in 112 participants in the Bogalusa Heart Study (Kelly et al., 2016). High risk participants had lower microbial diversity, as well as an increase in the abundance of *Prevotella* and *Tyzzerella*, and a decrease in *Alloprevotella* and *Catenibacterium*. A study in a cohort of 405 Chinese subjects found an enrichment in *Enterobacteriaceae* and *Streptococcus* spp. in atherosclerosis patients, suggesting that at least some of the associations between the gut microbiome and CVD might be population-specific (Jie et al., 2017).

The mechanisms by which the microbiome plays a role in CVD are however still poorly understood. One possible mechanism is the production of bacterial metabolites that induce the differentiation of pro- and anti-inflammatory cytokines, which has been demonstrated to be of critical importance in mouse models of colitis (Atarashi et al., 2013). Alternatively, the gut microbiota has also been shown to contribute to atherosclerosis through the conversion of choline or L-carnitine into TMAO (trimethylamine-*N*-oxide) (Koeth et al., 2013). Plasma TMAO is a biomarker of CVD risk associated with increased atherosclerotic stenosis, risk of major cardiovascular events, and mortality (Senthong et al., 2016). The deleterious effects of TMAO are hypothesized to be due to its promotion of platelet aggregation (Ridaura et al., 2013; Romano et al., 2015; Kreznar et al., 2017; Chen et al., 2018).

Given the reported associations between microbiome and CVD, several groups have investigated whether the beneficial effects of PA in CVD risk could be partially mediated by the changes induced in microbial composition. A study of professional rugby athletes and matched controls found that athletes had lower inflammatory status, and enrichment in bacterial diversity (Clarke et al., 2014). Differences were also observed in the abundance of 48 bacterial taxa, with notable enrichment in *Ruminococcaceae*, *Succinivibrio*, and *Akkermansia* in athletes compared to controls. However, diet was significantly distinct between the groups, and so the differences in microbial diversity and composition might be attributable both to PA and nutritional intake.

A more recent study aimed at disentangling the correlation between exercise, diet, and obesity status in shaping the gut microbiome. Eighteen lean and 14 obese subjects underwent 6 weeks of supervised endurance-based exercise training, followed by a washout period of 6 weeks in which they returned to a sedentary lifestyle (Allen et al., 2018b). Interestingly, changes in gut microbiome were dependent on obesity status, and short-chain fatty acids concentrations increased in lean but not in obese participants. Additionally, gut microbiome alterations disappeared once exercise ceased, suggesting that sustainment of PA is required for these changes to persist.

The causality of these associations is difficult to assess in human studies. Germ-free (GF) animals provide a suitable model to determine whether exercise-mediated changes in gut microbiome can induce specific phenotypes. Allen et al. (2018a) showed that transplanting the gut microbiome from either exercised or not exercised mice to GF mice induced specific distinct changes in the microbiome, metabolome, colonic inflammation, and body mass of the recipient mice (Allen et al., 2018a). Furthermore, colonization from exercised mice resulted in an attenuated response to DSS-induced colitis. These results demonstrate that exercise can alter gut microbiome and that those changes can result in beneficial health outcomes for the host.

Overall, current evidence points to an association between CVD and gut microbiome through the production of bacterial metabolites that induce potent host pro- or anti-inflammatory responses. Strategies to alter bacterial content are therefore of high interest for their potential therapeutic value. Together with diet (Llewellyn et al., 2017) and fecal transplants (Paramsothy et al., 2017), PA represents an additional approach through which the beneficial effects of gut microbiome modulation can be achieved.

## Conclusion

**FIGURE 1:**
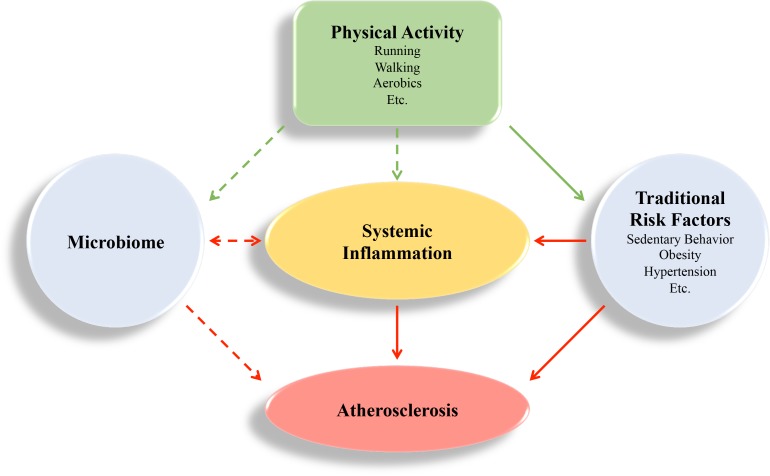
Visual Summary. Atherosclerosis is an inflammatory disease that is regulated by systemic inflammation. Physical activity can have beneficial effects (green arrows) by regulating systemic inflammation directly, and through behavioral and environmental factors that cause inflammation, like sedentary behavior, obesity, hypertension, and the microbiome. These risk factors contribute to deleterious effects (red arrows) and promote atherosclerosis development. The impact of the microbiome on atherogenesis is an emerging field, and the mechanism of how physical activity regulates the microbiome is not completely understood. Inflammation may also regulate the microbiome (van den Elsen et al., 2017; Chen et al., 2018).

Ongoing research focusing on the immune system and CVD continues to probe the molecular intricacies that contribute to atherosclerosis, including the individual contribution of immune cells, cytokines, and the microbiome (summarized in **Figure 1**). While an association between systemic inflammation, gut microbiome, and CVD is emerging, there is a great need to further our understanding of what constitutes normal biological variation versus pathological changes.

Immune modulatory therapeutics like Canakinumab show great promise to aid in the treatment of atherosclerosis, yet, the development of molecularly targeted pharmacological intervention is a challenging process due to the multifactorial nature of atherosclerosis. Additionally, the use of drugs to control atherosclerosis can be very costly posing a growing economic burden on the society. PA has proven to be a general modulator of systemic inflammation with some emerging effects on the gut microbiome that may be beneficial to overall cardiovascular health. While more research is needed to understand the implications of these changes, improving healthy behaviors by incorporating PA as part of a healthy lifestyle is a promising way to combat CVD.

## Author Contributions

DF, JC, and CG were responsible for writing and editing the manuscript.

## Conflict of Interest Statement

The authors declare that the research was conducted in the absence of any commercial or financial relationships that could be construed as a potential conflict of interest.
